# Artemisinin

**DOI:** 10.3201/eid2007.ET2007

**Published:** 2014-07

**Keywords:** etymologia, artemisinin, quing hao, sweet wormwood, antimalarial drug, malaria, China

## Artemisinin [ahrʺtə-misʹĭ-nin]

Artemisinin is an antimalarial lactone derived from *qing hao* (*Artemisia annua* or sweet wormwood). The medicinal value of this plant has been known to the Chinese for at least 2,000 years. In 1596, Li Shizhen recommended tea made from *qing hao* specifically to treat malaria symptoms. The genus name is derived from the Greek goddess Artemis and, more specifically, may have been named after Queen Artemisia II of Caria, a botanist and medical researcher in the fourth century bce.
